# WU Polyomavirus in Patients Infected with HIV or Hepatitis C Virus, Connecticut, USA, 2007

**DOI:** 10.3201/eid1507.090150

**Published:** 2009-07

**Authors:** Michael A. Miller, Carla Weibel, David Ferguson, Marie L. Landry, Jeffrey S. Kahn

**Affiliations:** Yale University School of Medicine, New Haven, Connecticut, USA

**Keywords:** Polyomavirus, WUPyV, KIPyV, HIV, hepatitis C virus, viruses, Connecticut, USA, dispatch

## Abstract

WU polyomavirus (WUPyV) was detected in 10 (8.3%) of 121 HIV-positive plasma specimens, 0 (0%) of 120 HIV-negative serum specimens, and 2 (2.5%) of 79 hepatitis C virus (HCV)–positive serum specimens. KI polyomavirus was not detected in HIV-positive plasma or HCV-positive serum specimens. HIV-infected persons may be susceptible to systemic WUPyV infection.

In 2007, 2 new human polyomaviruses, KI polyomavirus (KIPyV) and WU polyomavirus (WUPyV), were identified. KIPyV was initially detected in an extract obtained from 20 pooled randomly selected nasopharyngeal aspirates, and WUPyV was detected in a nasopharyngeal aspirate from a 3-year-old child from Australia who had a diagnosis of pneumonia (*1*,*2*). These viruses have since been detected in respiratory tract specimens from symptomatic and asymptomatic children, although no clear association with respiratory disease has been demonstrated (*3*–*6*).

Previously identified human polyomaviruses (BK virus [BKV] and JC virus [JCV]) cause clinical disease in immunocompromised persons (*7*,*8*). Although viremia may be associated with immunosuppression, correlation of JCV DNA in peripheral blood with development of progressive multifocal encephalopathy in AIDS patients remains controversial (*9*). BKV DNA has been detected in blood of renal transplant patients, and BKV load may be predictive of polyomavirus-associated nephropathy (*10*).

## The Study

The pathogenesis and clinical spectra of WUPyV and KIPyV, particularly in immunocompromised persons, have not been defined. To investigate whether WUPyV or KIPyV is present in persons with chronic viral infection and perhaps compromised immunity, we conducted a cross-sectional study in which we screened the following for WUPyV and KIPYV DNA: plasma samples from HIV-infected persons, serum samples from hepatitis C virus (HCV)–infected persons, and a control group of HIV-negative persons.

Three groups of samples submitted to the Clinical Virology Laboratory at Yale–New Haven Hospital in 2007 were screened: HIV PCR-positive plasma, HCV PCR-positive serum, and HIV antibody-negative serum. Patient identifiers were removed and these specimens were tested as part of our ongoing investigation for newly identified viruses. Collection of specimens and clinical data was approved by the Yale University Human Investigation Committee and was compliant with Health Insurance Portability and Accountability Act regulations.

Nucleic acids were extracted from each specimen by using QIAamp nucleic acid purification kits (QIAGEN, Valencia, CA, USA). Screening for WUPyV DNA has been described (*6*). Briefly, we performed an initial PCR screening specific for the virus capsid protein 2 (VP2) gene by using primers described by Gaynor et al. (*2*) and a nested PCR (Table 1, primers 1 and 2). To confirm results, DNA from all PCR-positive samples was reextracted and screened with primers specific for the region of the genome containing the noncoding control region (NCCR), which includes the virus origin of replication (genome coordinates 5213 to nt 36 of the circular viral genome). This screening included an initial PCR (Table 1, primers 3 and 4) and a nested PCR (Table 1, primers 5 and 6). The nested PCR generated a 328-bp amplicon. Screening for KIPyV DNA by PCR included a nested PCR specific for the VP1 gene according to the protocol described by Allander et al. (*1*). Positive and negative controls were included in each set of PCRs.

**Table 1:** Primers used in PCR to detect WU polyomavirus in serum specimens, Connecticut, USA, 2007

Primer	Name	Sequence (5′ → 3′)	Genome coordinates
1	WU2F*	GCGCATCAAGAGGCACAGCTACTATTTC	1377–1400
2	WU2R*	GCGCCTAGCCTGTGAACTCCATC	1510–1528
3	WUoriFnest†	CTCATTTCCCCCTTTGTCAGGATG	5011–5034
4	WUoriRII†	CTTTCCGCTGGACTACAAAGGGC	317–339
5	WUoriF†	GTAAATTTCCCCAGCAGGTC	5075–5095
6	WUoriR†	CGGAAACTTTAAAAGGTCACAG	153–174

*Primers used by Wattier et al. (*6*). †The amplicon generated with these primer spans across nt 1 of the 5,229-bp circular virus genome.

All PCR products were sequenced by using 377 DNA automated sequencers (Applied Biosystems, Foster City, CA, USA) at the W.M. Keck Biotechnology Resource Laboratory at Yale University School of Medicine. For WUPyV, phylogenetic analysis was performed on a 194-bp fragment within the amplified region of NCCR (nt 5197 to nt 159 of the circular viral genome) by using Lasergene MegAlign software (DNASTAR Inc., Madison, WI, USA) (ClustalW alignment method). The only clinical data available for these deidentified serum specimens were HIV/HCV status and virus loads. HIV and HCV virus loads were determined in the Clinical Virology Laboratory by quantitative reverse transcription–PCR using commercially available diagnostic tests. The Fisher exact test was used to determine whether the difference in the percentage of WUPyV-positive specimens in HIV-positive and HIV-negative patients was statistically significant.

Ten (8.3%) of 121 HIV-positive specimens and 0 (0%) of 120 HIV-negative samples were positive for WUPyV (p<0.01) (Table 2). Two (2.5%) of 79 HCV-positive samples were positive for WUPyV. One of the 10 WUPyV-positive, HIV-positive specimens was also positive for HCV. Current HIV status for 2 of the WUPyV-positive, HCV-positive persons was not available. None of the HIV-positive or HCV-positive specimens screened were positive for KIPyV (Table 2). HIV-negative specimens were not screened for KIPyV.

**Table 2:** Detection of WU and KI polyomaviruses in HIV-positive plasma, HIV-negative serum, and HCV-positive serum specimens, Connecticut, USA, 2007*

Specimen group	No. WU virus–positive specimens/total no. specimens tested (%)	No. KI virus–positive specimens/total no. specimens tested (%)
HIV+	10/121 (8.3)†	0/120
HIV–	0/120 (0)	Not tested
HCV+	2/79 (2.5)	0/80

*HCV, hepatitis C virus. †p<0.01 vs. HIV– samples.

Mean HIV loads for WUPyV-positive and WUPyV-negative, HIV-positive persons were 32,200 copies/mL (range 2,930–88,300 copies/mL, median 25,100 copies/mL) and 131,500 copies/mL (range 509–750,000 copies/mL, median 59,600 copies/mL), respectively. Mean HCV loads for WUV-positive and WUV-negative persons were 1,302,200 copies/mL (range 88,600–3,906,600 copies/mL, median 1,909,000 copies/mL) and 3,091,000 copies/mL (range 2,030–32,800,000 copies/mL, median 1,150,000 copies/mL), respectively.

Amplified sequences from WUPyV-positive serum specimens were compared with available sequences from GenBank (all of which were obtained from respiratory specimens). Several nucleotide polymorphisms were observed in the amplified portion of the NCCR of the New Haven WUPyV serum isolates (Figure). None of the nucleotide changes in the New Haven strains were mapped to the 4 putative large T-antigen binding sites within the origin of replication (*2*).

**Figure:**
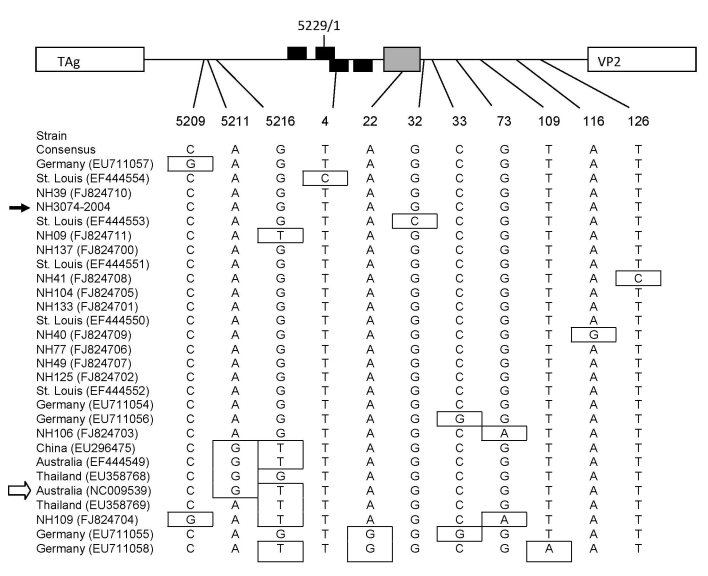
Nucleotide polymorphisms in the noncoding region of WU polyomavirus (WUPyV), Connecticut, USA, 2007. Sequences spanning nt 5197 to 159 of the circular viral genome of New Haven human serum isolates and all available sequences from GenBank (all from respiratory specimens) were subjected to phylogenetic analysis. A map of the noncoding region within the viral genome is indicated at the top of the figure (not to scale). The arbitrary last (5229) and first (1) nucleotide of the circular viral genome are indicated. The putative large T antigen (TAg) binding sites for each strand of the double-strand genome are indicated by black boxes, and the A/T-rich region is indicated by a gray box. Position of nucleotide polymorphism is indicated below the map. Strains (location and GenBank accession nos.) are listed in order according to phylogenic analysis. The consensus sequence is listed first, and polymorphisms are indicated by boxes. The strain indicated by the black arrow is a WUPyV New Haven respiratory isolate (*6*), and the strain indicated by the white arrow is the original WUPyV isolate (*2*). VP2, virus capsid protein 2.

## Conclusions

We detected WUPyV in plasma specimens from HIV-infected persons and in serum specimens from HCV-infected persons in Connecticut in 2007. Detection of WUPyV indicates that this newly identified polyomavirus is present in peripheral blood of HIV-positive or HCV-positive persons. It does not appear that WUPyV viremia correlates with HIV or HCV virus load. We did not assess the WUPyV serologic status of these persons. However, if the seroepidemiology of WUPyV is similar to that of BKV and JCV, we predict that >85% of the persons screened will have detectable antibodies to WUPyV (*11*). Whether antibody status for WUPyV correlates with viremia is unknown. Viremia may represent primary infection or reactivation of latent infection.

Viremia has been described for JCV and BKV. JCV DNA in serum/plasma may correlate with the degree of immunosuppression. However, blood from viremic persons infected with JCV has a low positive predictive value for development of progressive multifocal encephalopathy in AIDS patients (*9*). A recent study suggested that screening for BKV replication is useful in identifying patients at risk for BKV-associated nephropathy, which may enable early interventions such as renal biopsy and reduction of immunosuppression (*12*). However, because of the lack of clinical data available for WUPyV-positive persons in our study, it was not possible to make any clinical correlations.

The absence of KIPyV in HIV-positive or HCV-positive peripheral blood specimens suggests that host susceptibility for KIPyV may differ from that of WUPyV, as for JCV and BKV. However, this hypothesis was not supported by a recent study that reported KIPyV and WUPyV in autopsy lymphoid tissues of AIDS patients (*13*). Whether WUPyV or KIPyV cause disease in HIV-positive persons or other populations remains to be determined.

Our data demonstrate that WUPyV was detected in peripheral blood of HIV- and HCV-infected persons. However, the scope of this study was limited because clinical data were not available for study participants. Whether WUPyV or KIPyV have oncogenicity or other pathogenicity in immunocompromised hosts remain to be determined. The role of polyomaviruses in human cancers has been extensively investigated but conclusive evidence is lacking (*14*). The genome of Merkel cell polyomavirus, a new polyomavirus, was found to be integrated within the cellular genome of Merkel cell carcinoma tissue samples, which suggests a role for this virus in a specific tumor (*15*). Therefore, studies to assess the oncogenic potential of WUPyV and KIPyV are also needed.
